# The search for the “next” euphoric non-fentanil novel synthetic opioids on the illicit drugs market: current status and horizon scanning

**DOI:** 10.1007/s11419-018-0454-5

**Published:** 2018-11-28

**Authors:** Kirti Kumari Sharma, Tim G. Hales, Vaidya Jayathirtha Rao, Niamh NicDaeid, Craig McKenzie

**Affiliations:** 1 Fluoro Agro Chemicals Division, CSIR-Indian Institute of Chemical Technology, Hyderabad, 500007 India; 2 AcSIR-IICT, CSIR-Indian Institute of Chemical Technology, Hyderabad, 500007 India; 3 Division of Systems Medicine, School of Medicine, Ninewells Hospital University of Dundee, Dundee, UK; 4 Forensic Drug Research Group, Centre for Anatomy and Human Identification, School of Science and Engineering, University of Dundee, Dundee, UK; 5 Leverhulme Research Centre for Forensic Science, School of Science and Engineering, University of Dundee, Dundee, UK

**Keywords:** Novel synthetic opioids, Psychoactive substances, U-drugs, Bromadol, Clinical and forensic toxicology

## Abstract

**Purpose:**

A detailed review on the chemistry and pharmacology of non-fentanil novel synthetic opioid receptor agonists, particularly *N*-substituted benzamides and acetamides (known colloquially as U-drugs) and 4-aminocyclohexanols, developed at the Upjohn Company in the 1970s and 1980s is presented.

**Method:**

Peer-reviewed literature, patents, professional literature, data from international early warning systems and drug user fora discussion threads have been used to track their emergence as substances of abuse.

**Results:**

In terms of impact on drug markets, prevalence and harm, the most significant compound of this class to date has been U-47700 (*trans*-3,4-dichloro-*N*-[2-(dimethylamino)cyclohexyl]-*N*-methylbenzamide), reported by users to give short-lasting euphoric effects and a desire to re-dose. Since U-47700 was internationally controlled in 2017, a range of related compounds with similar chemical structures, adapted from the original patented compounds, have appeared on the illicit drugs market. Interest in a structurally unrelated opioid developed by the Upjohn Company and now known as BDPC/bromadol appears to be increasing and should be closely monitored.

**Conclusions:**

International early warning systems are an essential part of tracking emerging psychoactive substances and allow responsive action to be taken to facilitate the gathering of relevant data for detailed risk assessments. Pre-emptive research on the most likely compounds to emerge next, so providing drug metabolism and pharmacokinetic data to ensure that new substances are detected early in toxicological samples is recommended. As these compounds are chiral compounds and stereochemistry has a large effect on their potency, it is recommended that detection methods consider the determination of configuration.

## Introduction

Novel psychoactive substances (NPS) are continually evolving and emerging onto the illicit drugs market; however, the rate of emergence appears to be slowing down. Many compounds appear and disappear rapidly and it is challenging to attempt to carry out detailed research on all of them. It is, therefore, necessary to take a risk assessment and harm reduction based approach to focus on those substances with the greatest prevalence and propensity for harm. Materials containing fentanyl and fentanils (fentanyl analogues) are considered by many the greatest concern due to their prevalence, potency and diversity [1–3]. There are also other non-fentanil novel synthetic opioids (NSOs) emerging across the illicit synthetic drug market, which adapt and diversify in a manner that is responsive to developments in the illicit drug supply chain and the new national and international legislation designed to disrupt them [4]. Many of these NSOs are currently, or have previously been, present on the illicit market and have appeared in drug seizure casework or have been detected in body fluids submitted to clinical or forensic toxicology laboratories, albeit with low prevalence [5]. Some are being openly discussed on drug user fora and are available for sale on darkweb or clearweb vendor sites [6–8] and may not have been detected within forensic and clinical samples [9, 10]. Many of these substances were originally developed as potential research targets in the pharmaceutical industry, their synthesis routes published and patented, and their pharmacological properties studied using the methodologies available at the time [11–23]. In most, if not all, cases their development was halted before clinical trials were carried out, often due to the presence of unwanted pharmacological side effects. Their emergence onto the illicit drug market is actively and effectively tracked by national and international early warning systems such as the European Monitoring Centre for Drugs and Drug Abuse (EMCDDA) early warning system (EWS) and this coordination ensures vital and rapid information sharing and risk assessment [24].

This review focusses on a sub-group of NSOs known colloquially as the “U-compounds” or “U-drugs”. These substances were originally synthesized in the 1970s and 1980s by the Upjohn Company, a pharmaceutical company from Kalamazoo, MI, USA and the review takes in other related compounds synthesized around the same time within the company and by other research teams [11–23].

The Upjohn Company, along with many other pharmaceutical companies at the time, were attempting to find a non-addictive opioid analgesic that did not cause respiratory depression or dependency, with a similar potency to morphine, a global search which continues to this day. This work, although not producing commercially viable opioid analgesics at the time, produced potent and, in some cases, highly selective opioid receptor agonists that have helped to unravel the mode of action and specific opioid receptor binding properties of the opioid family of drugs.

A pattern of the emergence of these substances onto the illicit drugs market has become apparent in recent years. Early papers and patents are scoured for compounds of interest; identified substances (or closely related compounds with small structural alterations) then appear for sale on vendor sites, often marketed as “legal” alternatives to now controlled substances; the substances are either provided as free samples to buyers of other substances or purchased directly by users (who are often referred to as “psychonauts”) and their effects discussed and reviewed in online drug user fora. The most prevalent of these non-fentanil NSOs so far has undoubtedly been U-47700 (*trans*-3,4-dichloro-*N*-[2-(dimethylamino)cyclohexyl]-*N*-methylbenzamide), described by users as giving rise to highly desirable, apparently short-lived euphoric effects creating a desire to redose [25]. U-47700 has recently been controlled at the national and international level, most notably in China [26, 27], and so the illicit market appears to be seeking alternative compounds to appeal to users of U-47700.

It is difficult to predict which compounds may appear on the illicit drug market and which, if any, will become popular with users and/or gain a more substantial foothold in the mainstream illicit drug market. Now that U-47700 is internationally controlled and production and export from China is prohibited, some users will be seeking a “legal” alternative to other controlled opioids with a lower potency than the fentanils, whilst others may be seeking a substance which mimics the effects of U-47700 irrespective of its legal status. The laboratories synthesising the drugs will look to past structure activity relationship (SAR) studies, structural adaptations that appeared successful with other unrelated drugs classes or to those analogues which are the easiest and cheapest to produce due the availability of precursor compounds and the ease of synthesis.

Many jurisdictions react in a responsive way to the appearance of such novel substances on the illicit drugs market rather than in a pre-emptive or predictive manner. In doing so they could inadvertently be driving the structural diversification of the substances appearing on the illicit market.

## Licit drug development: evolution of the *N*-substituted benzamide analgesics and related compounds

### Allen and Hanburys Limited compounds (AH-compounds)

**Fig. 1:**
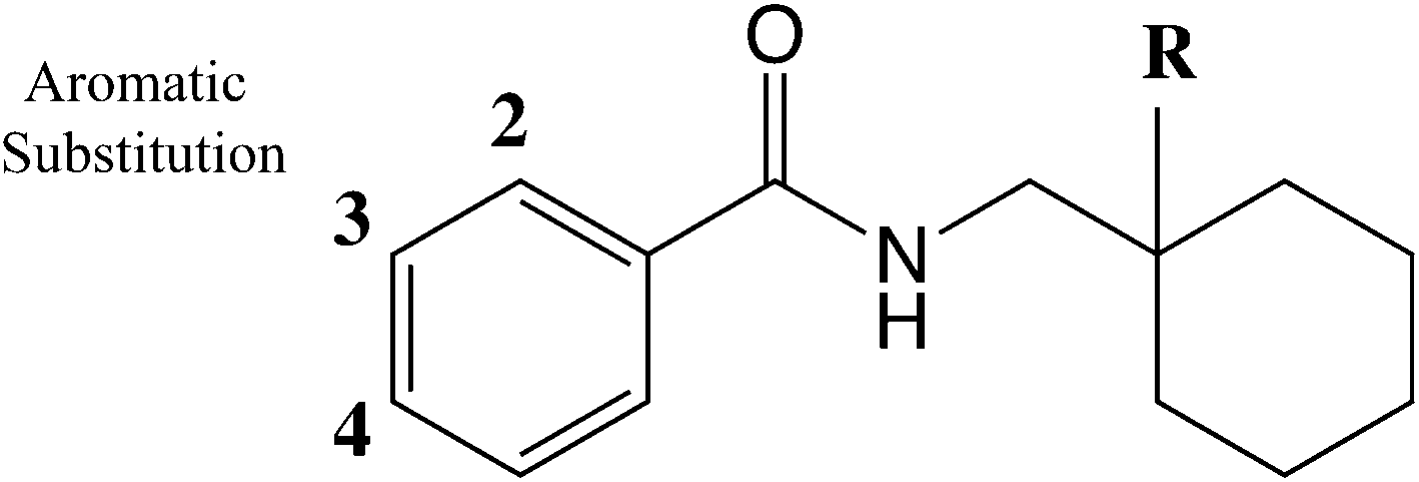
Allen and Hanburys Limited candidate opioid analgesic compounds generic structure

The antinociceptive effects of the *N*-substituted cyclomethylbenzamides were explored by Brittain et al. [17] at the United Kingdom pharmaceutical company, Allen and Hanburys Limited, in collaboration with the University of Aston, Birmingham, UK. The company had been acquired by Glaxo in 1958, but retained its name until 1978 after which it was subsumed into Glaxo Group Research Ltd. A series of compounds were synthesized and tested in vivo in mice. Harper et al. [11] published details of the synthesis of a series of 60 cyclohexyl derivatives (1-(3,4-dichloro-benzamidomethyl)cyclohexyl-dimethylamines). Thirty-five of these were subjected to preliminary pharmacological screening. Tests were carried out at oral doses of 100 mg/kg (behaviour, body temperature, antimaximal electroshock, antagonism of leptazol-induced convulsions, and effects on phenylquinone-induced writhing); oral doses of 50 mg/kg (tremor, hypothermia); and a subcutaneous dose of 100 mg/kg (hot-plate effects observed directly and on interaction with morphine). The *N*-substituted benzamide derivatives, identified as compounds 51-53, 57 and 58 in the paper [11], some of which were also reported in the Brittain et al. paper referred to using their “AH” codes [17], were identified as the most promising compounds (see Fig. 1 for the generic structure and Table 1 for structural details). One compound in particular, 3,4-dichloro-*N*-[1-(dimethylamino)-cyclohexyl]methylbenzamide, named AH-7921, was identified as having the greatest analgesic properties, approximately 0.8 times that of morphine in comparative studies, and was selected for further acute animal studies. This work, including detailed synthesis methods, was submitted as a United States patent in 1974, assigned to Allen and Hanburys Limited, the patent being granted in 1976 [28]. During these studies, there was evidence of the addictive potential of AH-7921, with physical dependence observed in the animals tested and so trials were discontinued at that time [17]. Further animal testing was carried out by the Glaxo Group Research Ltd, later confirming that undesirable effects (e.g. Straub tail response, hypothermia, sedation, respiratory depression and inhibition of gastrointestinal propulsion) occurred at concentrations similar to those required for analgesia confirming that this compound should not be selected for further study [29].

**Table 1:**
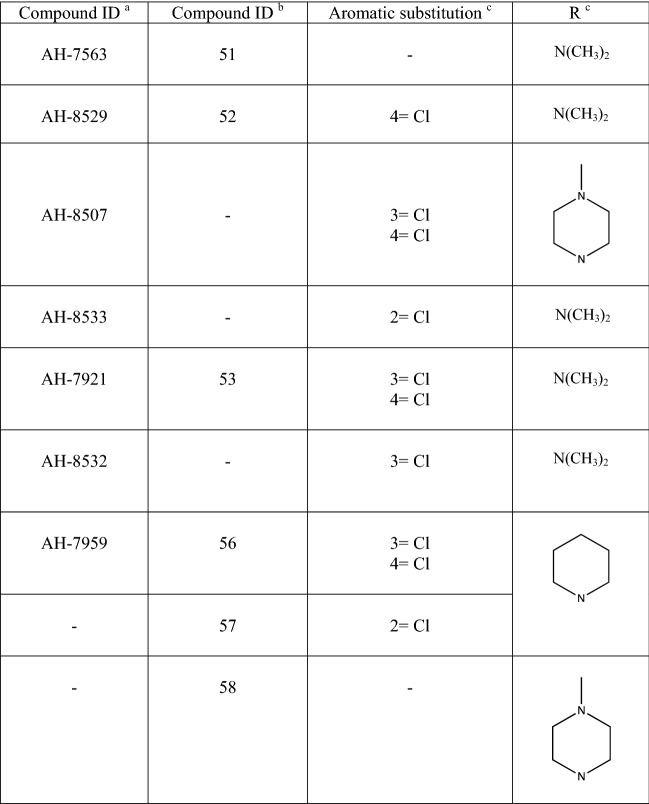
Identification and structural features of selected opioid analgesics developed by Allan and Hanburys Limited

^a^As identified in [17]

^b^As identified in [11]

^c^Refer to Fig. 1 for the generic molecular structure for these analogues

### Upjohn Company compounds (U-compounds or U-drugs)

**Fig. 2:**
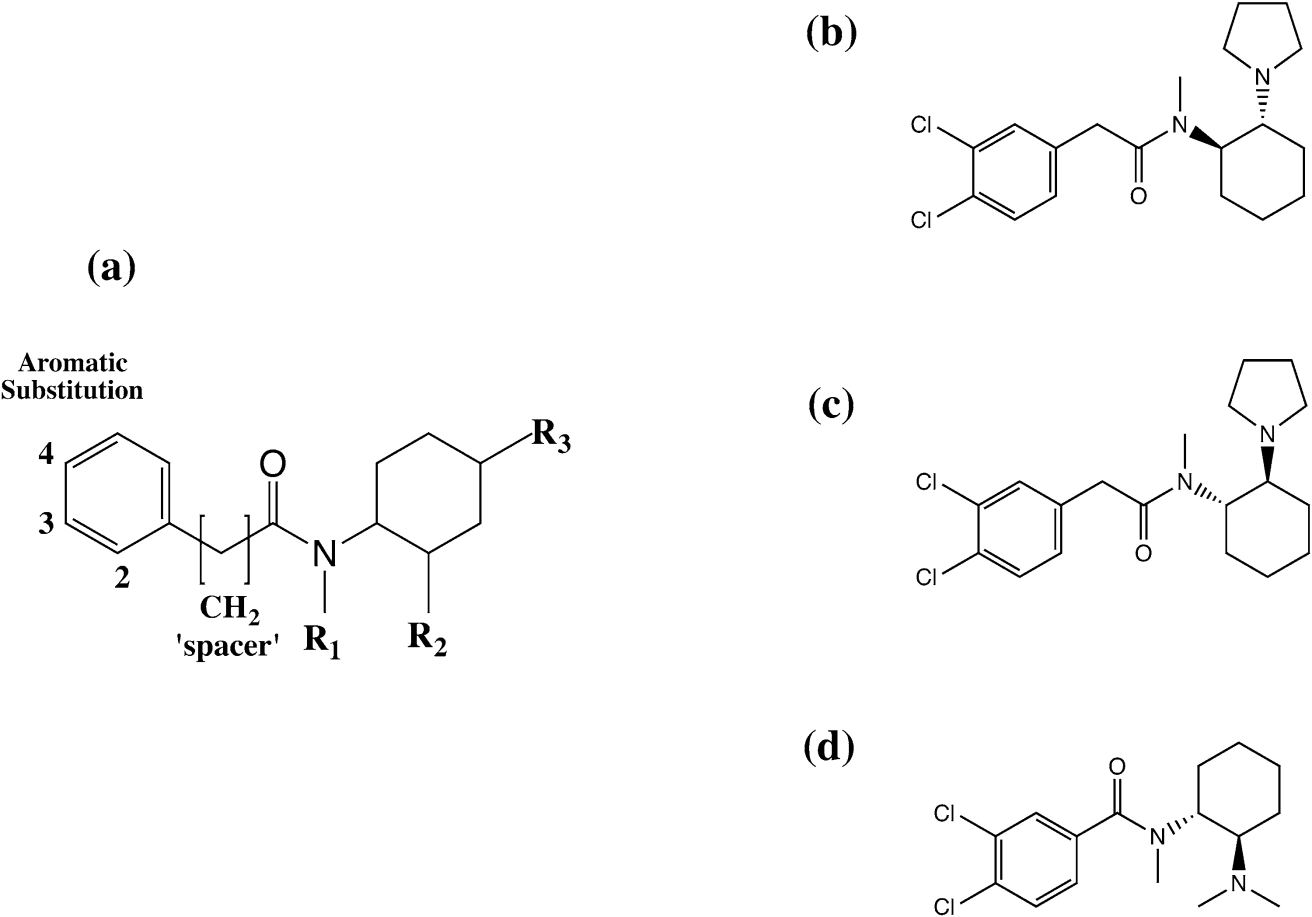
Upjohn Company *N*-substituted benzamides and acetamides (U-drugs); **a** generic structure, **b** (+) *trans*-U-50488; 2-(3,4-dichlorophenyl)-*N*-methyl-*N*-((1*R*,2*R*)-2-(pyrrolidin-1-yl)cyclohexyl)acetamide, **c** (−) *trans*-U-50488, also known as U-50488H; 2-(3,4-dichlorophenyl)-*N*-methyl-*N*-((1*S*,2*S*)-2-(pyrrolidin-1-yl)cyclohexyl)acetamide, **d** (+) *trans*-U-47700; (3,4-dichloro-*N*-((1*R*,2*R*)-2-(dimethylamino)cyclohexyl)-*N*-methylbenzamide)

*N*-*Substituted benzamides and acetamides* From 1973, chemists at the Upjohn Company synthesized a series of *N*-(2-aminocycloaliphatic)benzamide compounds leading, in 1975, to the discovery of the selective kappa-opioid receptor (KOR) agonist, U-50488 (*trans*-2-(3,4-dichlorophenyl)-*N*-methyl-*N*-(1*S*,2*S*)-2-(pyrrolidin-1-yl)cyclohexyl)-acetamide), Fig. 2. The (−) *trans*-(1*S*,2*S*) isomer, also referred to as U-50488H, had greater affinity for KOR than the (+) *trans*-(1*R*,2*R*) or either of the *cis*-configurations or the (±) *trans*-racemic mixture, highlighting the need to take into account the important effect of stereochemistry on their pharmacological behaviour [15]. A large number of related compounds were patented in 1978 [12] and further related patents followed [e.g. 30, 31]. Information on some of these compounds is provided in Table 2. A number of different methodologies to determine in vitro opioid receptor binding affinity (*K*_D_) have been used in the cited literature and care should be taken when comparing such data directly between studies. Perhaps a more useful measure of comparison between studies is the relative affinity of the test compound to the different opioid receptors. Binding affinity to a target receptor is of itself not a direct measure of “potency” and such methods do not take into account off-target pharmacological effects which may affect downstream biological effects and toxicity.

**Table 2:**
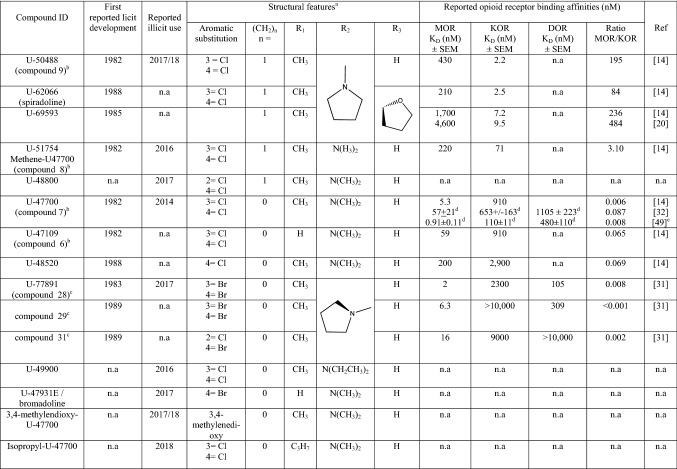
Identification, structural features and pharmacological data of selected *N*-substituted benzamides and acetamide opioid analgesics (colloquially known as U-drugs’) developed by the Upjohn Company

MOR, KOR and DOR refer to the μ-, κ- and δ-opioid receptors respectively

*n.a* data not available, *SEM* standard error of the mean, *K*_*D*_ dissociation constant/ligand-receptor affinity

^a^Refer to Fig. 2a for the generic molecular structure

^b^Refers to compound identification in [12]

^c^Refers to compound identification in [31]

^d^In vitro receptor binding data provided is the inhibition constant (*K*_*i*_) rather than *K*_D_ [31]

^e^Data from Janowsky (2016) as cited in [49, 50]

A detailed description of U-50488H was first published in the peer reviewed literature in 1982 [18, 19]. It did not cause respiratory depression and was not habit-forming, but was found to cause sedation, diuresis and dysphoria, the latter properties being undesirable in medicinal products and indicative of KOR-mediated effects. The (−) *trans*-(1*S*,2*S*) U-50488 (U-50488H) and the (±) *trans*-racemic mixture are commercially available as reagents from specialist chemical supply companies (e.g. Merck, Tocris Bioscience, Axon Medchem etc.) and are widely used as model KOR agonists in experimental studies [15]. The discovery and deconvolution of the structure activity relationships of U-50488H, and its related compounds, including the important role of stereochemistry in agonist activity, was elegantly described by the Upjohn chemist responsible for its development, Jacob Szmuszkovicz [15]. The generic structure of the *N*-substituted benzamides and acetamides is shown in Fig. 2a. The structural requirements for a potent KOR agonist appear to be a combination of the following: (1) a (−) *trans*-(1*S,2S*) configuration; (2) the presence of an *N*-methyl group on the nitrogen adjacent to the carbonyl and (3) the presence of a methylene (CH_2_) “spacer” [15]. This is further illustrated by the modifications of the U-50488H structure which lead to the synthesis of two further highly selective KOR agonists with a (−) *trans*-(1S,2S) configuration; U-62066 (spiradoline, 2-(3,4-dichlorophenyl)-*N*-methyl-*N*-[(5*R,*7*S,*8*S*)-7-pyrrolidin-1-yl-1-oxaspiro[4.5]decan-8-yl]acetamide) and U-69593 (*N*-methyl-2-phenyl-*N*-[(*5*R*,7*S*,8*S)-7-pyrrolidin-1-yl-1-oxaspiro[4.5]decan-8-yl]acetamide) both of which have a heterocyclic ring structure fused to the cyclohexyl moiety in the R_3_ position (Fig. 2a, Table 1) [20–22].

The properties of U-50488H were compared to those of the assumed novel mu-opioid (MOR) agonists U-47109 (3,4-dichloro-*N*-(2-(dimethylamino)cyclohexyl)benzamide), U-47700 and U-51754 (2-(3,4-dichlorophenyl)-*N*-[2-(dimethylamino)cyclohexyl]-*N*-methyl-acetamide) (Fig. 2) to demonstrate the drug’s selectivity for the KOR [13]. In this study, U-47700, which has a (+) *trans*-(1*R*,2*R*) configuration (and it is assumed U-47109 and U-51754 also have the same configuration), was found to have 7.5, eight and three times greater analgesic effect than morphine in antinociceptive tests (tail flick, tail pinch and hydrochloric acid writhing respectively). U-50488H, U-51754 and U-47109 were found to be less potent than morphine, but still had measurable analgesic effects [13]. Characteristic MOR mediated effects (Straub tail, arched back, increased locomotor activity) were observed when mice were treated with U-47700, U-51754 and U-47109 but were not observed for U-50488H.

Further deconvolution of the “U-drug” structure activity relationships to MOR and KOR was reported in 1988 [14]. The removal of a chlorine atom in the meta-position on the phenyl moiety of U-47700 to give U-48520 (4-chloro-*N*-(2-(dimethylamino)cyclohexyl)-*N*-methylbenzamide) leads to an apparent decrease in MOR affinity and further removing the chlorine in the para-position and replacing it with a hydroxyl group leads to a complete loss of affinity to both MOR and KOR (Table 2). The presence of the methylene bridge in the structure appears to decrease affinity to MOR and increase affinity to KOR (Table 2).

Opioid receptor binding profiles and the antinociceptive properties of further related compounds were investigated in 1989 by Fujimoto et al. [32] including U-77891 (*trans*-3,4-dibromo-*N*-methyl-*N*-[1-methyl-1-azaspiro[4.5]decan-6-yl]benzamide) (Table 2). This compound was first described in an Upjohn Company patent from 1985 [30] and is an *N*-substituted tertiary amide containing a heterocyclic ring structure on the cyclohexyl moiety, an *N*-methyl group adjacent to the carbonyl and halogen atoms in the 3,4-positions of the aromatic ring (bromines instead of chlorines of U-50488/U-47700), but unlike U-50488 and U-51754 no methylene spacer is present. Other closely related analogues (referred to as compounds 29-32 in the paper) are also described. The lack of the methylene spacer (and possibly a (+) *trans*-(1*R*, 2*R*) configuration—although this is not specifically mentioned) in the structures of U-77891 and compounds 29-32 appears to produce highly selective MOR agonists. The presence of halogens in the 3,4-position of the aromatic ring appears to enhance activity and is likely to protect the aromatic moiety from enzymatic hydroxylation once the substance is consumed. The selective MOR agonist properties of U-47700 were further confirmed in a more recent study [33]. Despite the development of a wide range of related compounds in later patents, U-47700 has remained one of the substances in this class with the greatest reported analgesic potency and greatest MOR binding.

**Fig. 3:**
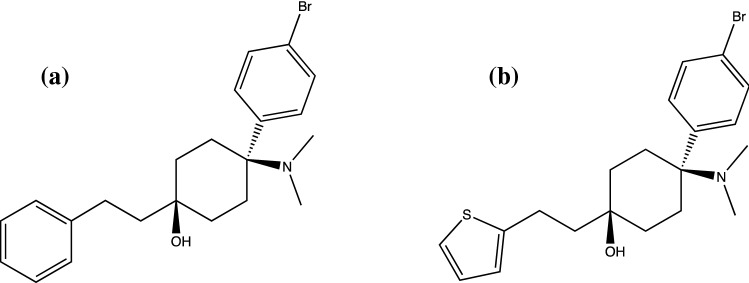
Aminocyclohexanol based analgesics **a** BDPC/bromadol and **b** C8813

*4*-*Aminocyclohexanols* Around the same time that the *N*-substituted benzamide and acetamide compounds were being developed at the Upjohn Company, a patent was filed in 1979 and published in 1982 for a series of 4-aminocyclohexanol compounds [34]. *Trans*-4-(*p*-Bromophenyl)-4-(dimethylamino)-1-phenethylcyclohexanol, referred to simply as “compound 1” in the original research paper (BDPC, also referred to as bromadol in online drug user discussion fora and vendor sites) was found to be a highly potent analgesic in animal studies (tail flick, tail pinch, hydrochloric acid writhing), effects that were reversed by naloxone, indicating that the analgesic activity was mediated via the MOR [35]. The *trans* isomer was determined to be considerably more potent than the *cis* isomer (Fig. 3), the molecular structure being found to be superimposable upon the molecular structure of the potent synthetic opioid fentanyl [35].

It was originally stated that BDPC/bromadol had 10,000 times the analgesic potency of morphine in animal studies [34] although a more recent study, comparing analgesic potency using a standard mouse hot plate assay where BDPC/bromadol was introduced by intraperitoneal injection, suggests that its analgesic potency *may* be lower, at around 500 times that of morphine and 2.9 times that of fentanyl [36]. BDPC/bromadol was the lead compound for a further series of 4-amino-4-arylcyclohexanone analogues and their derivatives prepared by the Upjohn team to provide structure activity relationship data [16, 37, 38]. None had analgesic properties greater than BDPC/bromadol, although a *trans*-chlorinated analogue showed similar potency as did an analogue where the phenethyl moiety was replaced with a cyclohex-3-ene moiety [37]. Another analogue of BDPC/bromadol, *trans*-4-(*p*-bromophenyl)-4-(dimethylamino)-1-(2-thiophen-2-yl-ethyl)-cyclohexanol (C8813, thiobromadol Fig. 3.) was synthesized and characterized in 2003 [36]. This work was the first to study receptor binding affinities for both BDPC/bromadol (*K*_*i*_ = 1.49 nM for MOR) and C8813 (*K*_*i*_ = 1.37, 3.24 and > 1000 nM for MOR, DOR and KOR respectively). The antinociceptive potency observed previously and in the same study is perhaps greater than that expected given the opioid receptor binding affinity data presented and so warrants further detailed study. As far as the authors are aware, none of these substances have been subject to clinical trials and none have been developed commercially, although a method for the purification of BDPC/bromadol after synthesis was published by a Chinese research team in 2014 and refers to a “commercially available bromadol” solution bought from a Chinese vendor [39].

## The appearance of non-fentanil novel synthetic opioids on illicit drug markets

**Fig. 4:**
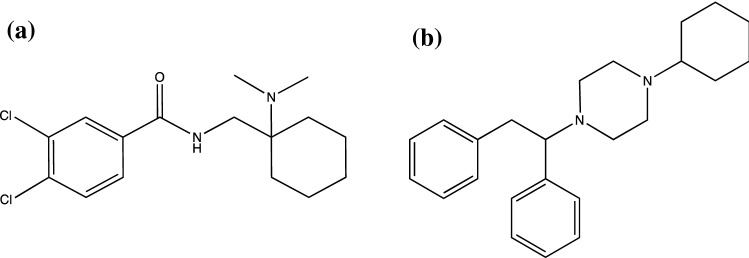
Internationally controlled non-fentanil novel synthetic opioids **a** AH-7921 and **b** MT-45

The first non-fentanil novel synthetic opioid to appear on illicit markets was the opioid analgesic, AH-7921 around 2012–13 (Fig. 4a), subsequently controlled internationally in 2014 and in the UK in 2015 as a Class A drug under the Misuse of Drugs Act [40–42]. A structurally unrelated compound, MT-45 (1-cyclohexyl-4-(1,2-diphenylethyl)piperazine, Fig. 4b), originally synthesized and studied in Japan in the 1970s as a candidate opioid analgesic, emerged and was controlled under the same legislation soon after [43–45]. A fluorinated analogue, 2F-MT-45 (1-cyclohexyl-4-(1-(2-fluorophenyl)-2-phenylethyl)piperazine) has recently been detected in the UK, however there is no evidence that the substance is available on vendor sites, possibly due to the unpopularity of MT-45 (and it’s recorded side effects) amongst users [46]. U-47700 (Fig. 2d), a structural isomer of AH-7921, was first detected in Europe in Sweden in late 2014. AH-7921, MT-45 and U-47700 have already been the subject of a number of detailed reviews and risk assessments [4, 5, 41, 44, 47–54] and all have now been internationally controlled. U-47700 was made a Schedule 1 substance in the United States in November 2016 [55] and the UK controlled it as a Class A Schedule 1 substance under the Misuse of Drugs Act 1971 and Misuse of Drugs Regulations 2001 in June 2017 [56]. China, widely agreed to be the main production site, banned its manufacture and export, along with MT-45, in July 2017 [27].

Since its appearance on the illicit drug market, U-47700 has become one of the most prevalent non-fentanil NSOs, particularly in the United States, and case reports referring to the substance in drug seizures and toxicology cases continue to be published [57–76]. In Europe, U-47700 was reported to the EMCDDA EWS in January 2015 [77]. The reporting of the presence of U-47700 in products that users may naturally believe to contain a different class of drug is of particular concern. U-47700 was recently detected in plant/incense-like products in Belgium, which would normally be expected to contain synthetic cannabinoid receptor agonists (SCRAs), colloquially known as “spice” [78]. In early 2016, U-47700 was detected in approximately 3000 illicit tablets of at least four visually distinct types in Scotland, one of which was visually identical to those known to normally contain the benzodiazepine etizolam and two of which would likely be described as “street diazepam” by users, illicit tablets with a high prevalence in the illicit drug market in Scotland [79].

As a result of the international control of U-47700 and the ban on its manufacture in, and export from, China [27], it is perhaps predictable that U-47700 prevalence will in the future, or will have already, started to decline as has been previously observed for many, but not all, substances in the same situation. U-47700 analogues originating from, or adapted from compounds reported in the early literature, have started to appear on illicit drug market and a high state of vigilance and information sharing is maintained through international early warning systems. Summary structural information on the compounds emerging on to the illicit market is provided in Table 2.

U-49900 (3,4-dichloro-*N*-[2-(diethylamino)cyclohexyl]-*N*-methyl-benzamide) is a novel structural analogue of U-47700 (Table 2), first noted on “research chemical vendors” websites and on online drug user fora in November 2016 [80]. A test purchase and analytical characterization study was carried out by the European Union (EU) co-funded RESPONSE project, run by the Slovenian National Forensic Laboratory in November 2016 [81], and soon after an alert was published by the EMCDDA EWS [82]. Unlike U-47700, for which at least limited early (and now updated) pharmacological data exist, no such data for this analogue are currently available. The chemical structure retains the majority of the key features that were identified by Loew et al. [14] as promoting MOR agonist mediated effects (3,4-dichloro on the phenyl ring, the lack of a methylene spacer and an *N*-methyl group adjacent to the carbonyl), but with *N*-diethyl substitution on the cyclohexyl moiety instead of the *N*-dimethyl substitution of U-47700 (Table 2). Fabregat-Safont et al. [83] identified and analytically characterized U-49900 in great detail using a drug sample seized in Spain. In addition, a monograph on U-49900 was recently published by the Scientific Working Group on Drugs (SWGDRUG) [84]. U-49900 has since been reported in seizures and toxicological samples in the United States. Krotulski et al. [85] describe the identification of U-49900 in post-mortem blood and urine specimens collected after an apparent drug overdose and U-49900 detection has also been reported in seized drug samples by the United States Drug Enforcement Administration (DEA) [86].

U-51754 was described in the original Upjohn Company patents and related studies [12–14] and has recently appeared on illicit drug vendor sites listed as both methene-U-47700 (most likely named as such due to the presence of the methylene spacer and to illustrate its relationship to U-47700 for marketing purposes) and U-51754 (see Table 2). U-51754 was first detected in Europe via a test purchased sample received by the RESPONSE project in October 2016. The sample was analysed and reported in January 2017 [87] and the data shared via the EMCDDA EWS [88]. U-48800 (2-(2,4-dichlorophenyl)-*N*-(2-(dimethylamino)cyclohexyl)-*N*-methyl-acetamide), differing from U-51754 only in the positions of one of the chlorine atoms on the phenyl moiety (Table 2), was not included in the original Upjohn Company patents or any of the related pharmacological studies. U-48800 was detected in Germany in a sample received for testing in October 2017 by the ADEBAR project, a project co-funded by the Internal Security Fund of the EU, the German Federal Criminal Police Office, the German Federal Customs Service and a number of State Bureaus of Criminal Investigations. The data was reported to the RESPONSE project in February 2018 [89] and detection of U-48800 in seized samples was also notified by the DEA in the last quarter of 2017 [86].

In early animal studies U-51754, was reported to be approximately ten times less potent as a MOR agonist (and therefore approximately 0.75 times the analgesic potency of morphine) than U-47700. It is likely to have more pronounced KOR mediated effects [13] than U-47700 due to the presence of a methylene bridge in the molecular structure [14], a feature shared by U-48800 for which no in vitro or in vivo data are currently available.

*U-77891* (Table 2) started to appear on user discussion fora in early 2016 [90] continuing into 2017 [91] although there are no available reports of users actually receiving or taking the substance. It has appeared on clearweb vendor sites, although there is no confirmation that it is actually being sold and to the best of the author’s knowledge no seizures of this compound have been reported.

*U-50488* (Table 2) has been commercially available from specialist chemical suppliers for some time and many research studies have utilised this model KOR agonist in experimental studies. The first reported illicit seizure in Europe was in Sweden in November 2017 and was reported to the EMCDDA EWS [92]. Analysis was carried out by the National Forensic Centre, Sweden and U-50488 was identified by comparison with a commercially available (±) *trans* isomer reference standard, although no determination of the stereochemistry of the U-50488 in the seized sample was carried out (Simon Dunne, National Forensic Centre, Linköping, Sweden, personal communication). As mentioned in the previous sections, the (−) *trans*-(1*S*,2*S*) isomer is a selective KOR agonist, whilst the (+) *trans*-(1*R*,2*R*) isomer is a non-selective opioid receptor agonist with relatively poor affinity for both the MOR and KOR receptors [15].

*U-47931E/bromadoline* (*trans*-4-Bromo-*N*-[2-(dimethylamino)cyclohexyl]-benzamide; Table 2) is a MOR agonist referred to in the original 1978 Upjohn Company patent [12] as example 10, with the name bromadoline being registered by the company in 1983 [93]. A 1985 study referred to bromadoline as a compound undergoing “clinical testings in humans for safety and efficiency” [94]. The paper refers to further studies being carried out on the disposition kinetics of bromadoline in humans and canines; however, apart from being included in a further study by Glaxo Group Research Ltd in 1987, the authors have been unable to find any further published studies on this compound [95]. Around September 2017, U-47931E/bromadoline appears to have started to be sold on web vendor sites and was identified for the first time in Europe by the RESPONSE project via a test purchased sample received in October 2017 from a web vendor [96]. The analytical data for the substance was reported in November 2017 and an EMCDDA EWS notification was communicated soon after [97]. The stereochemistry of the sample was not determined and, as far as the authors are aware, no seizures have yet been reported in the professional or peer-reviewed literature. A reference standard has been produced for the U47931E/bromadoline analogue *N*-methyl-U-47931 and is now commercially available [98], however, to the authors’ knowledge there is currently no indication that this compound has yet appeared on the illicit drugs market.

*Isopropyl-U47700* (*trans*-3,4-dichloro-*N*-[2-(dimethylamino)cyclohexyl]-*N*-methyl-benzamide; Table 2) is one of the most recent U-drug analogues to appear on web vendor sites and was identified by the RESPONSE project via a test purchased sample, received in January 2018 [99]. To date the stereochemistry of the sample has not been determined and no European seizures have been reported in the open literature. Isopropyl-U47700 has been reported in toxicological samples from two cases submitted in March 2018 in the United States in which 3,4-methylenedioxy-U-47700 was also detected [9] and was reported for the first time in seizures in the DEA emerging threat report for the 2nd quarter of 2018 [100].

*3,4-Methylenedioxy-U-47700* (trans-*N*-2-(dimethylamino)cyclohexyl)-*N*-methylbenzo[d] [1, 3] dioxole-5-carboxamide; Table 2) is the latest compound of this series to be reported to the EMCDDA EWS having been detected in a single sample seized in Poland in April 2018 [101]. The stereochemistry of the sample was not determined. The two chlorines in the 3, 4-position of the benzene ring have been replaced by a 3,4-methylenedioxy ring system, similar to that seen in a large number of psychoactive substances including substituted phenethylamines and cathinones. In early structure activity relationship studies for the *N*-substituted benzamides and acetamides, the removal of the chlorine atoms in the 3,4-positions of the benzene ring led to a complete removal of MOR mediated effects however there is no information available on the effect on the pharmacological profile of such a structural alteration. There is no information on the pharmacological effects of this substance although the substance has been detected in five cases submitted from January 2018 in the United States, and in two of these, isopropyl-U47700 was also detected [9, 10].

*BDPC/bromadol* To the best of the authors’ knowledge, at the time of writing there has only been one report of a drug seizure involving BDPC/bromadol. The seizure was reported in Montreal, Canada in 2013, although analytical data confirming the presence of the drug or details of the amounts seized is not currently publicly available and therefore the report cannot be confirmed [102]. There are no publicly available reports of any seizures in Europe.

### Online discussion forum based discussions of emerging U-drugs and BDPC/bromadol

There are a number of drug-related internet fora and online drug user communities. The benefits and risks of the use of data from such fora for novel illicit drug research purposes have been discussed previously [103, 104]. When using such sites, users are generally seeking reports of drug use and experiential information and will also post self-reports on the characteristics of novel drugs and their subjective experiences with them. Previous studies have identified four main themes of the discussion threads on such fora where users sought (1) reliable information on novel and emerging substances, (2) dosage and administration information, (3) information on subjectively experienced effects of novel and emerging substances, and (4) support and safety information from other users [103]. Online communities such as this have been previously described as having a self-regulating approach to novel drugs appearing on the illicit market, rapidly sharing information on either harms associated with novel substances or if subjective effects are positive or negative. Researchers seeking information from such fora must acknowledge that the motivation for users to post information about new substances cannot be verified and the actual substance taken by the user can also not be verified—a user may not have been sold the substance they believe they have been sold by a web vendor. In addition, researchers must be aware of the placebo effect with regards to a self-reported subjective experience; the fact that users may have used other substances before, during or after the substance under test; and as regular drug users, they are likely to have built up tolerances to, in this case, opioids which will affect their response to a particular substance. Nevertheless, some useful information can be obtained on the timeline of the appearance of new drugs on the market and users subjective experience of them. The information provided below gives examples of online discussion fora threads only and is not an exhaustive list of such discussions available online.

Online discussions on U-49900 have already been reported previously [83] and are not discussed in detail here. Postings referencing either U-51754 or its alternative name, methene-U-47700, began to appear between May and September 2016 indicating that the drug had appeared on web vendor sites. One user referred to U-51754 as an alternative to U-47700 after the latter had “become illegal” and predicted that it might be dysphoric rather than euphoric [105]. Another user reported taking the drug rectally and claimed the effects were strong, dysphoric and dissociative and with a relatively short duration of around 45 min [106]. Forum posts in another discussion thread, discuss the original research published by the Upjohn Company [107]. Other users reported unpleasant dissociative experiences with no euphoria [108]. The reaction from forum users is generally negative and advice to avoid the substance has been communicated. Postings referencing U-48800 began to appear in April 2017 [109, 110]. In general, the response to this drug by users of online fora has been, as for U-51754, generally negative, although some posts have indicated a more positive, but mild experience [110]. User fora discussions of U-47931E/bromadoline started to appear around September 2017 and appear to identify variability in the products sold as U-47931E/bromadoline on vendor websites and also indicate that vendors were providing samples for testing with other purchases. In early posts, at least two distinct batches of the drug were discussed, one white in colour and one brown/tan. Detailed user reports for both batches describe distinct subjective user experience differences between the two [111, 112]. Insufflation of the brown/tan sample allegedly produced a rush and euphoria whilst another user states that the subjective effects of U-47931e/bromadoline are milder than for U-47700, but last longer [113, 114].

Discussions relating to 3-4-methylenedioxy-U47700 appeared in May and June 2017 [115, 116] and for isopropyl-U-47700 appear to start around November and December 2017 with users stating the latter drug had been offered for sale on vendor sites [117, 118]. The user response to these substances appears almost uniformly negative.

*BDPC/bromadol* has been discussed on online user fora for many years [e.g. 119, 120]; however, substances purporting to be BDPC/bromadol seem to have recently increased in prevalence on vendor sites (early to mid 2017). Since then a number of experiential reports have been posted and a common theme of such user reports is the lack of the sought after euphoric effects [8, 121]. Some users posting on a German language forum have reported buying BDPC/bromadol as a purported 10 mg/mL solution [121]. Due to its reported analgesic potency in animal studies, its reported MOR affinity, its appearance on vendor sites in a number of forms and the potential for harm, the prevalence of this compound in drug seizures and toxicological samples should be closely monitored.

It seems clear, at least from the limited information provided from drug user forum posts, that novel U-drug analogues and BDPC/bromadol are appearing on web vendor sites, although they may appear and disappear over relatively short time periods based on availability and user demand or response. Their actual prevalence in casework and appearance in biological samples is mostly unknown, but would be expected, at this time, to be very low. In general, the majority of these compounds, in the reports available, have been viewed negatively relative to the user experiences relating to U-47700. Given an understanding of the structure activity relationships and known pharmacology of those U-drug analogues that have been studied, this may be seen as not overly surprising. It will be interesting to observe if users will grow tired of trying these subjectively unpopular analogues or if they will continue to experiment with new analogues as they enter the market in the hope of a more subjectively positive user experience.

## Toxicology, metabolism and toxicovigilance

When a drug is consumed it may or may not undergo biotransformation to more polar metabolites to aid its excretion from the body and those metabolites may themselves be pharmacologically active. If biotransformation is rapid and exhaustive, it may be challenging for toxicologists to identify the parent drug in urine and blood samples, especially if the intoxication was not fatal and samples were taken a considerable time after drug ingestion. It is therefore important to understand metabolic processes including actual and intrinsic drug clearance rates and metabolite identification. This is especially true if analysts wish to preemptively scan for substances, and their postulated metabolites, that may enter the illicit drug market and may appear in biological matrices submitted to the toxicology laboratory for testing.

For U-47700, *N*-desmethyl-U-47700 and *N,N*-didesmethyl-U-47700 were identified both in in vitro assays using human liver microsomes (HLM) and casework samples as the main (major) metabolites of U-47700 with additional hydroxylation occurring at varying positions on the cyclohexyl moiety [59–62, 122].

For U-49900, *N*-desethyl-U-49900 and *N,N*-didesethyl-U-49900 were identified as the major metabolites, with, as for U-47700, *N*-dealkylation occurring on the *N*-cyclohexyl moiety, as well as minor metabolites with subsequent hydroxylation on the cyclohexyl ring. Unlike U-47700 *N*-demethylation also occurred subsequently on the nitrogen adjacent to the carbonyl. These metabolites were determined in vitro using HLM [122].

There are close structural similarities between the U-drug analogues entering the illicit drug market. From the data available for U-47700 and U-49900 it is possible to predict the metabolites formed from the novel substances discussed in this review. An early study on the metabolism of U-47931E/bromadoline appears to confirm this, with two metabolites proposed to be *N*-desmethyl-U-47931E and *N,N*-didesmethyl-U-47931E detected in blood, plasma and urine from human and canine samples [95]. It is recommended that the pseudo-molecular ions of the parent drug and predicted metabolites for the novel non-fentanil synthetic opioids discussed in this paper are incorporated into high resolution mass spectrometry screening methods in toxicology laboratories and that these are updated as and when in vitro and in vivo metabolite data become available.

A large number of studies have reported the identification of U-47700 in toxicological samples and information on pharmacokinetics is starting to be published. Koch et al. [123] reported the analysis of samples from a patient who was admitted to hospital following the alleged smoking of an opioid. Serum and urine samples were taken and flubromazepam, its metabolite hydroxyl-flubromazepam and U-47700 were detected. Serum samples were taken over a known time period and concentrations of U-47700 decreased exponentially from an initial serum concentration of 370 ng/mL. The half-life of U-47700 in this patient was calculated to be approximately 6 h.

Concentrations of U-49900 in toxicological samples have only been reported in one casework sample to date which involved a fatal intoxication [85]. Postmortem blood and urine concentrations of U-49900 were found to be 1.5 and 2.2 ng/mL, respectively, and tetrahydrofuranylfentanyl (399 ng/mL blood) and methoxy-phencyclidine (1.0 ng/mL blood) were also detected.

Studies on the bioavailability, pharmacokinetics and metabolism of BDPC/bromadol have yet to be published.

## Legislative controls for non-fentanil novel synthetic opioids

A number of jurisdictions are reacting to the appearance of new non-fentanil NSOs in the illicit drugs market. In the United States, U-47700 is a Schedule 1 substance under the Controlled Substances Act and therefore its analogues will be controlled under the Controlled Substance Analogue Act of 1986. In July 2017, the state of North Carolina controlled U-47700, U-49900 and U-77891 under the State Synthetic Opioid and Other Dangerous Drugs Control Act [124]. In March 2018, the state of Minnesota added bromadol to schedule 1 of the controlled substances law [125]. Canada recently controlled U-47700 and “its salts, derivatives, isomers and analogues, and salts of derivatives, isomers and analogues”, including U-47931E/bromadoline, U-47109, U-48520, U-50211 and U-77891 [126]. In addition, the legislation controlled the precursor of U-47700, and many of its analogues, *N*^1^,*N*^1^,*N*^2^-trimethylcyclohexane-1,2-diamine and its salts. The precursor and synthetic route was described in the original Upjohn Company patents and related scientific papers, although other synthetic routes using alternative precursors are likely. In April 2018, Sweden brought into force a legislative ban on U-47931E/bromadoline to join AH-7921, MT-45 and U-47700 [127]. In Latvia, U-51754 was controlled by the Center for Disease Prevention and Control in February 2017 [128]. In the UK, U-47700 is the only U-drug currently covered by the Misuse of Drugs Act 1971 and the Misuse of Drugs Regulations (2001), whereas all other psychoactive analogues and the aminocyclohexanes would be controlled by the Psychoactive Substances Act 2016. In China, U-47700 is the only U-drug thus far to be controlled and so the production of analogues and further market development may continue if there is seen to be a demand for such substances. The legislative controls introduced in China are highly impactful, greatly decreasing international availability of such substances and are to be commended. Controlling analogues of U-47700 and including BDPC/bromadol in future drug control legislation may be an effective and pre-emptive response and may halt the diversification of such analogues entering the market currently and in future.

## Predicting the next emerging U-drugs on the illicit drugs market

The introduction of new U-drug analogues onto the illicit market has appeared, up to now, to be relatively haphazard with compounds deriving either from the original Upjohn Company patents and the related peer reviewed scientific literature (U-51754, U-47931E, U-77891, U-50488) or from untested structural adaptations of those original compounds (U-48800, U-49900, isopropyl-U47700, 3,4-methylenedioxy-U47700). In general, these substances do not appear as prevalent as U-47700 has been and are generally poorly received, if reports on novel drug user fora are to be considered representative. As drugs with similar pharmacological effects to U-47700 may continue to be sought, it should be considered a possibility that other analogues, aimed at preserving the key structural features of U-47700, e.g. promoting selective MOR mediated effects, or alternatively new derivatives with little regard for the original structure activity relationships will appear on the illicit market. Some additional substances have recently been reported on vendor sites, MPF-47,700 (advertised as a U-47700 analogue) and U-4TDP (advertised as being similar to U-51754) but these cannot be verified as novel substances as no chemical structures are provided and to date no web-sourced materials have been characterised or their chemical structures elucidated [129].

## Conclusions

For all of the drugs reviewed, it is important that the effect of stereochemistry on their pharmacological profile is not underestimated. From a legal perspective this may be of little interest as the analyst must simply determine that a certain substance is present or not, and determine whether or not it is controlled under the relevant national and international legislation; however, it is important from a toxicological and harm reduction perspective. Such information is also likely to provide information on synthetic routes and precursor sources. Determination of stereochemistry is rarely, if ever, included in current standard analytical testing protocols for illicit drug seizures.

There is a requirement to determine the chemical structures of metabolites of new substances to ensure their timely detection in clinical and forensic toxicology casework, aiding the monitoring of their emergence as substances of abuse. This is particularly true for BDPC/bromadol for which no published data currently exists. It is important to have a full understanding of what vendors and users seek to gain from the development of such novel illicit substances and as a result to pre-empt adverse events and/or predict new substances as, or before, they emerge. As a result, drug testing, clinical and forensic toxicology laboratories further increase the effectiveness of national and international monitoring systems. In our opinion, where there is a potential future risk, reference materials should continue to be synthesized in both a responsive and pre-emptive manner; web-sourced materials should be analytically characterized; their metabolism studied in vitro and the identity and pharmacological activity of parent drugs and metabolites elucidated.
